# Association between the low-dose irinotecan regimen-induced occurrence of grade 4 neutropenia and genetic variants of *UGT1A1* in patients with gynecological cancers

**DOI:** 10.3892/ol.2014.2046

**Published:** 2014-04-08

**Authors:** HIROYUKI MORIYA, KATSUHIKO SAITO, NUALA HELSBY, SHIGEKAZU SUGINO, MICHIAKI YAMAKAGE, TAKERU SAWAGUCHI, MASAHIKO TAKASAKI, HIDENORI KATO, NAHOKO KUROSAWA

**Affiliations:** 1 Department of Pharmacy, Hokkaido Pharmaceutical University School of Pharmacy, Otaru, Japan; 2 Department of Molecular Medicine and Pathology, Faculty of Medical and Health Sciences, University of Auckland, Auckland, New Zealand; 3 Department of Anesthesiology, Sapporo Medical University School of Medicine, Sapporo, Japan; 4 Department of Pharmacy, National Hospital Organization Hokkaido Cancer Center, Sapporo, Japan; 5 Department of Gynecology, National Hospital Organization Hokkaido Cancer Center, Sapporo, Japan

**Keywords:** irinotecan, UDP-glucuronosyltransferase 1A1, polymorphism, neutropenia, gynecologic cancers

## Abstract

The occurrence of severe neutropenia during treatment with irinotecan (CPT-11) is associated with the *6 and *28 alleles of uridine diphosphate glucuronosyltransferase 1A1 (*UGT1A1*). However, the correlation between these variants and the occurrence of severe neutropenia in a low-dose CPT-11 regimen for the treatment of gynecological cancers has not been extensively studied. There are also no studies regarding the association between the 421C>A mutation in ATP-binding cassette sub-family G member 2 (*ABCG2*) and the occurrence of severe neutropenia in CPT-11-treated patients with gynecological cancers. The present study was designed to determine the factors associated with the occurrence of grade 4 neutropenia during chemotherapy for gynecological cancers with combinations of CPT-11 and cisplatin or mitomycin C. In total, 44 patients with gynecological cancer were enrolled in the study. The association between the absolute neutrophil count (ANC) nadir values, the total dose of CPT-11 and the genotypes of *UGT1A1* or *ABCG2* was studied. No correlation was observed between the ANC nadir values and the total dose of CPT-11. The ANC nadir values in the UGT1A1*6/*28 and *6/*6 groups were significantly lower compared with those in the *1/*1 group (P<0.01). Univariate analysis showed no association between the occurrence of grade 4 neutropenia and the *ABCG2* 421C>A mutation. Subsequent to narrowing the factors by univariate analysis, multivariate logistic regression analysis only detected significant correlations between the occurrence of grade 4 neutropenia and the UGT1A1*6/*6 and *6/*28 groups (P=0.029; odds ratio, 6.90; 95% confidence interval, 1.22–38.99). No associations were detected between the occurrence of grade 4 neutropenia and the heterozygous variant (*1/*6 or *1/*28) genotype, type of regimen or age. In conclusion, the UGT1A1*6/*28 and *6/*6 genotypes were found to be associated with the occurrence of severe neutropenia in the low-dose CPT-11 regimen for gynecological cancers. This finding indicates that the determination of *UGT1A1* variants may be as useful in CPT-11 chemotherapy for gynecological conditions as it is in colorectal and lung cancer patients treated with this drug.

## Introduction

Taxanes and platinum-containing agents are key drugs that are used in the chemotherapy for gynecological cancers. However, in Japanese patients who received optimal debulking surgery to treat stage II–IV clear-cell carcinoma of the ovary, an adjuvant chemotherapy regimen combining irinotecan (CPT-11) with cisplatin (CDDP) has been shown to prolong the progression-free survival time more than a regimen combining paclitaxel with platinum (1). In addition, a combination of CPT-11 and mitomycin C (MMC) has been reported to be effective in elderly Japanese patients with gynecological cancers who did not respond to the combination regimen of taxanes and platinum (2). Thus, although not currently used as the standard chemotherapy for gynecological conditions, regimens that include CPT-11 may have a useful role.

However, CPT-11 occasionally causes severe neutropenia. The inherited factors associated with the occurrence of this side-effect include genetic variants of uridine diphosphate glucuronosyltransferase 1A1 (*UGT1A1*), such as the *6 and *28 alleles (3–14). The risk of severe neutropenia is increased in individuals who are homozygous for the *28 allele (3–5,7). However, this association is not observed when CPT-11 is administered at a low dose for lung or colorectal cancer (9,15,16). Low-dose CPT-11 regimens, including CPT-11 + CDDP or CPT-11 + MMC, are used in certain gynecological cancers. However, associations between the occurrence of severe neutropenia and the UGT1A1*28 variant in these cases is not well known. By contrast, UGT1A1*6 is a variant found in the Asian population at a frequency higher than that of *28 (17–19). In Japanese patients with colorectal and lung cancer, the *6/*28 and *6/*6 genotypes are significantly correlated with the occurrence of severe neutropenia (7). This may indicate that these genotypes may be risk factors for CPT-11-induced severe neutropenia in Japanese patients with gynecological cancers. Furthermore, a study of the CPT-11 + CDDP regimen in gynecological conditions demonstrated that the risk of severe neutropenia is higher in patients with the *1/*6 genotype than in patients with the *1/*1 genotype (13). However, this study did not clarify the affect of the *6/*28 and *6/*6 genotypes on the risk of developing neutropenia.

In addition, the 421C>A (Q141K) variant of ATP-binding cassette sub-family G member 2 (*ABCG2*), which encodes the breast cancer resistance protein (BCRP), a transporter known to target various anticancer drugs, including CPT-11, has been reported to reduce the expression of *ABCG2* and cause resistance to CPT-11 *in vitro* (20). However, the 421C>A variant is not associated with CPT-11-induced severe neutropenia in patients with lung and colorectal cancer (8,21). Whether this single-nucleotide polymorphism (SNP) has any association with CPT-11-induced neutropenia in gynecological cancer is not known.

Thus, investigations of the associations between the occurrence of severe neutropenia during treatment with a low-dose CPT-11 regimen and these genetic variants in gynecological malignancies are, at best, incomplete. The present study was designed to clarify the role of these genetic factors in the occurrence of grade 4 neutropenia in patients treated with CPT-11 + CDDP or CPT-11 + MMC chemotherapy for gynecological cancer.

## Materials and methods

### Patients

The Institutional Review Board of the National Hospital Organization Hokkaido Cancer Center (Sapporo, Japan) approved the present study, and informed consent was obtained from all patients. Subjects were Japanese patients who received CPT-11-based chemotherapy in the Department of Gynecology, National Hospital Organization Hokkaido Cancer Center. The chemotherapy regimens used were CPT-11 + CDDP and CPT-11 + MMC. All the patients were evaluated to ensure they exhibited sufficient organ function, including bone marrow function, prior to beginning the regimens involving CPT-11. No patients were receiving drugs known to interact with CPT-11. The following were the exclusion criteria of this study: Previous CPT-11 administration, an Eastern Cooperative Oncology Group performance status of ≥3 and an age of <18 or >80 years old.

The clinical data, including the neutrophil count, of these patients were retrospectively investigated using information obtained from medical records. The absolute neutrophil count (ANC) nadir value was assessed during the first cycle of the regimen containing CPT-11. Severe neutropenia (grade 4) was determined using the Common Terminology Criteria for Adverse Events, version 3.0 (22).

### Genotyping

Genomic DNA was isolated from peripheral blood that was anticoagulated with K_2_-EDTA using a Puregene DNA Isolation kit (Qiagen, Hilden, Germany), according to the manufacturer’s instructions. The genotypes of the *UGT1A1* gene, including *6 and *28, were determined according to a previously described method (23). The presence of the 421C>A mutation in exon 5 of the *ABCG2* gene was determined by PCR, followed by sequencing. The primers used in the PCR and sequencing of this variant were synthesized by Sigma-Genosys Japan, Inc. (Ishikari, Japan). The sequences of the forward and reverse primers were 5′-GGTTCATCATTAGCTAGAACTTTAC-3′ and 5′-TGGAAAGCAACCATTTTTGA-3′, respectively. The PCR amplification was conducted using a PTC-200 pelitier thermal cycler (Bio-Rad Laboratories, Inc., Hercules, CA, USA) and AmpliTaq Gold® 360 Master Mix (Life Technologies, Inc., Carlsbad, CA, USA). The cycling conditions used were as follows: Initial denaturation at 95°C for 10 min, subsequent denaturation at 95°C for 30 sec, annealing at 58°C for 30 sec and primer extension at 72°C for 30 sec, repeated for 30 cycles, followed by a final extension at 72°C for 7 min. The *ABCG2* genotypes (421C>A) were determined by direct sequencing of the purified PCR products.

### Statistics

The Hardy-Weinberg equilibrium (HWE) test of the genotype frequency of *UGT1A1* and *ABCG2* in the subjects was conducted using Fisher’s exact test. Spearman’s rank correlation test was used to analyze the correlation between the total dose of CPT-11 and the ANC nadir values. Mann-Whitney’s U test with Bonferroni’s correction was applied for the comparison of the association of the genotypes of *UGT1A1* and *ABCG2* with the ANC nadir values. In the univariate analysis of the characteristics of the patients prior to chemotherapy, Mann-Whitney’s U test was applied to compare the values between grade 0–3 (G0-3) and grade 4 (G4) neutropenia groups. For the univariate analysis of the genotypes, previous treatments, regimens, type of cancer and performance status, Fisher’s exact test was applied to compare the values between the two groups. Variables with P<0.1 in these univariate analyses were then adopted as explanatory variables when conducting the multivariate logistic regression analysis, in which the incidence of G4 neutropenia was a dependent variable. The SPSS Statistics 21 software (IBM Japan Inc., Tokyo, Japan) and GraphPad Prism 5.0 (GraphPad Prism Software, San Diego, California, USA) were used for statistical analyses. A two-tailed value of P<0.05 was considered to indicate a statistically significant difference.

## Results

### Patient characteristics

A total of 44 patients (24 with ovarian cancer, 10 with endometrial cancer, 9 with cervical cancer and 1 with a tumor of the lower abdominal wall) were enrolled and evaluated during the period between July 2007 and September 2011. The patients received the following chemotherapy: Either 40–60 mg/m^2^ CPT-11 (on days 1, 8 and 15) and 40–60 mg/m^2^ CDDP (on day 1; n=22) or 70–150 mg/m^2^ CPT-11 (on days 1 and 15 or on days 1, 8 and 15) and 4–10 mg/m^2^ MMC (on day 1 or on days 1 and 15; n=22). In total, 10 patients developed G4 neutropenia (22.7%). The patient characteristics prior to chemotherapy are shown in Tables I and II.

### UGT1A1 and ABCG2 genotypes and allele frequencies

The number of patients with each genotype of *UGT1A1* was: *1/*1, n=25; *1/*6, n=3; *1/*28, n=9; *6/*28, n= 3; *6/*6, n=4; and *28/*28, n= 0. For the *ABCG2* 421C>A variant, there were 28 patients with the C/C genotype, 10 with C/A and six with the homozygous variant (A/A). No deviation from HWE was observed in the distribution of the genotypes of *UGT1A1* and *ABCG2* (P=0.204 and P=0.285, respectively). The allele frequencies of the polymorphisms were as follows: 0.159 for UGT1A1*6, 0.136 for UGT1A1*28 and 0.250 for 421A of *ABCG2*, which are similar to those previously reported in the Asian population (17–19).

### Association between CPT-11-induced neutropenia and the genotypes of UGT1A1 or ABCG2

**Figure 1:**
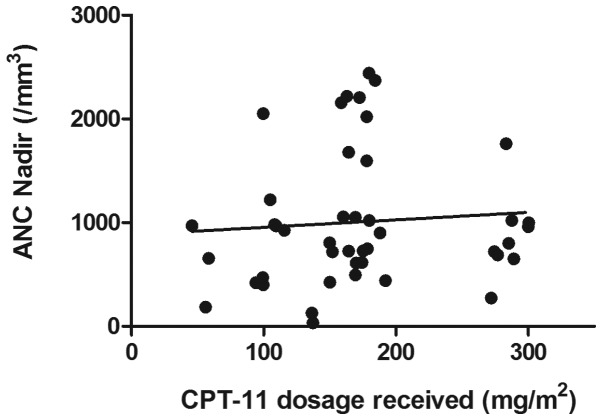
Correlation between the total dose of irinotecan (CPT-11) received in the first cycle and the absolute neutrophil count (ANC) nadir values (R^2^=0.006, P=0.185) in 44 patients with gynecological cancer treated with regimens containing CPT-11.

**Figure 2:**
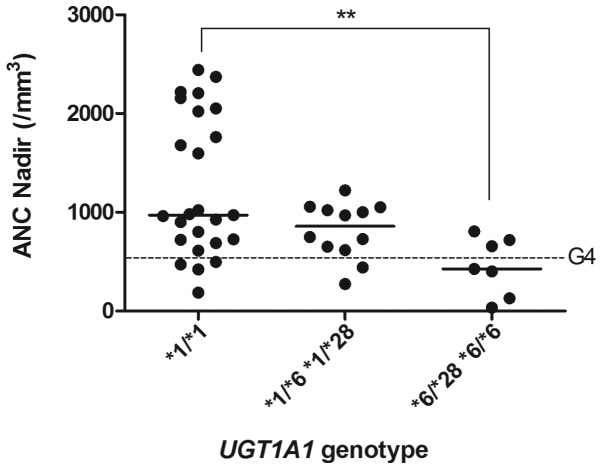
Correlation between the *UGT1A1* genotype and the absolute neutrophil count (ANC) nadir values during low-dose irinotecan (CPT-11) administration. The horizontal line for each genotype indicates the median of the ANC nadir values. The dotted line indicates the ANC at which grade 4 (G4) neutropenia is observed. ^**^P<0.01. *UGT1A1*, uridine diphosphate glucuronosyltransferase.

**Figure 3:**
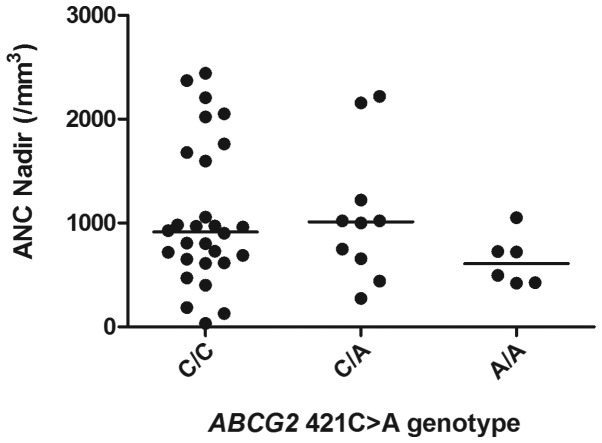
Correlation between the *ABCG2* (421C>A) genotype and the absolute neutrophil count (ANC) nadir values during low-dose irinotecan (CPT-11) administration. The horizontal line of ANC nadir values for each genotype indicates the median. *ABCG2*, ATP-binding cassette sub-family G member 2.

As shown in Fig. 1, no correlation was found between the total dose of CPT-11 in the first cycle and the ANC nadir values (R^2^=0.006, P=0.185). By contrast, comparison of the ANC nadir values among patients with each *UGT1A1* genotype revealed statistically significant differences between the *1/*1 group and the homozygous variant (*6/*28 or *6/*6) group (Fig. 2). No significant differences were observed for the other genotype pairs (Fig. 2). In addition, there were no significant differences in the ANC nadir values for any of the *ABCG2* 421C>A genotypes (Fig. 3).

### Associations of G4 neutropenia with patient characteristics and the genotypes of UGT1A1 and ABCG2

Investigation of the association between the incidence of G4 neutropenia and patient characteristics revealed a significant difference in pre-treatment liver function values for AST and ALT (P=0.018 and P=0.001, respectively; Table I), between patients with and without severe neutropenia. Although no significant difference in the incidence of G4 neutropenia was observed with age (P=0.098), patients developing this symptom appeared to be older.

A recessive model of inheritance best explained the significant difference in incidence of G4 neutropenia with respect to the *UGT1A1* gene (P=0.037; Table II). By contrast, there was no significant difference in either the dominant or recessive models of inheritance of the *ABCG2* 421C>A mutation and the risk of neutropenia.

There were no significant correlations between the incidence of G4 neutropenia with previous treatment, regimen, type of cancer or performance status. However, the CPT-11 and MMC combination regimen appeared to increase the incidence of G4 neutropenia compared with the combination of CPT-11 and CDDP (P=0.069).

Although liver enzyme function (AST, ALT and γ-GTP) was significantly different in patients with and without G4 neutropenia (P<0.1) in the univariate analysis, these factors were not used as explanatory variables in the logistic regression analysis, as all patients developing G4 neutropenia demonstrated values of these parameters that were within the normal range.

Multivariate logistic regression analysis was then used to confirm the significant association between the presence of the homozygous variant *UGT1A1* genotype (*6/*28 or *6/*6) and the risk of G4 neutropenia (P=0.029; odds ratio, 6.90; 95% confidence interval, 1.22–38.99). No significant differences were observed in the associations between the incidence of G4 neutropenia and the heterozygous variant genotype (*1/*6 or *1/*28), the type of regimen or the age of the patient.

## Discussion

The principal objective of the present study was to clarify the cause of severe neutropenia that occurred in the first cycle of a low-dose CPT-11 regimen in patients with gynecological cancer. A complicating factor was that the total dose of CPT-11 used in the present study varied from 40 to 150 mg/m^2^ among the patients. Since the variation in the total dose received may be associated with the occurrence of adverse reactions, it was first determined that there was no correlation between the total dose and the ANC nadir values. This indicates that the total dose of CPT-11 does not necessarily affect the ANC nadir values. To determine if the *UGT1A1* polymorphism is a factor, the association between the UGT1A1*6 and *28 genotype and the ANC nadir values in patients with gynecological cancers was investigated. This revealed that the patients with the homozygous variant (*6/*28 or *6/*6) had significantly decreased ANC nadir values and also that all the patients with these variants developed G3/4 neutropenia (i.e., a neutrophil count of <1000/mm^3^). This demonstrates the role of deficient UGT1A1 activity due to the presence of the homozygous variant genotype (UGT1A1*6/*28 or *6/*6) in the occurrence of severe neutropenia caused by the treatment of gynecological conditions with a low-dose CPT-11 regimen. However, the association of the UGT1A1*28/*28 genotype could not be investigated, as this genotype was not detected in the 44 patients studied. It has been previously reported that during high-dose CPT-11 chemotherapy, the ANC nadir values in the first cycle were significantly decreased in patients with the *28/*28 genotype (4). The UGT1A1*6 allele was not detected in this study of mainly Caucasian cancer patients. An investigation into the *28/*28 genotype and any decrease in the ANC nadir values in Japanese patients following low-dose CPT-11 treatment would require a large sample size due to the low-allele frequency of the *28 variant in the Japanese population.

It has been reported in Chinese patients with colorectal cancer that the incidence of CPT-11-induced G3/4 neutropenia is significantly higher in females than in males (24). Therefore, not only the differences in race, dose and regimen, but also the differences in gender should be considered when investigating associations between the ANC nadir values and the *UGT1A1* genotypes. In the present study, four patients who did not have variant alleles (*1/*1 genotype) developed G4 neutropenia. This indicates that factors other than *UGT1A1* genetic variation may be involved in the occurrence of severe neutropenia.

Multiple studies have indicated that high-dose CPT-11 regimens can be safely used in patients of ≥70 years of age (25–27). Although, it has also been demonstrated that the incidence of G3/4 neutropenia increases at ≥65 years of age (28). This indicates that the affect of aging on the risk of CPT-11-induced severe neutropenia requires scrutinization.

Additionally, in Japanese patients with colon and stomach cancer, the incidence of G3/4 neutropenia has been reported to be ~15% higher with CPT-11 + MMC compared with CPT-11 + CDDP (10). Thus, CPT-11 + MMC may increase the risk of severe neutropenia compared with CPT-11 + CDDP, but this is controversial.

In the univariate analysis of the present study, which was conducted as the initial investigation of these factors, age and regimen did indeed demonstrate a tendency to exert an effect on the risk of G4 neutropenia. However, the multivariate logistic regression analysis did not reveal a statistically significant association with age and/or regimen. Multivariate analysis demonstrated the involvement of only UGT1A1*6/*28 and *6/*6 as a risk factor for the occurrence of G4 neutropenia in patients with gynecological cancers who received low-dose CPT-11.

A previous study of Japanese patients with mainly lung and colorectal cancers reported that the risk of G3/4 neutropenia was significantly higher in patients with UGT1A1*6/*6 (10,12). In addition, the *6/*6 genotype is also reportedly involved in the occurrence of G4 neutropenia in Korean patients with non-small cell lung cancer (6,11). The data from the present study of patients with gynecological cancer also indicates a role for the UGT1A1*6/*6 genotype in neutropenia, similar to these previous studies of other types of cancers. In addition, the UGT1A1*6/*28 genotype has also been reported to increase the risk of CPT-11-induced G3/4 neutropenia in Japanese patients with colorectal or lung cancer (7,29), which agrees with the data from the present study on this genotype. By contrast, Gao *et al* (24) reported that there was no association between *6/*28 and G3/4 neutropenia in Chinese patients with colorectal cancer who received CPT-11. Such inconsistent associations indicate a necessity for further investigation of the UGT1A1*6/*28 genotype.

In a previous study that assessed the role of the heterozygous variant genotype, it was reported that UGT1A1*1/*6 and *1/*28 were not involved in the occurrence of G3/4 neutropenia in Japanese patients with colorectal cancer who had been treated with CPT-11 combined with 5-fluorouracil and leucovorin (30), which is similar to the results of the present study. However, in another study of Japanese patients with mainly lung or colorectal cancer (12), and also in a previous study of Japanese patients with gynecological cancers (13), the risk of G3/4 neutropenia in *1/*6 patients was demonstrated to be higher than that in *1/*1 patients. Thus, the associations between the heterozygous genotypes and the risk of neutropenia in Japanese patients are not consistent and require clarification.

The present study indicated that the *ABCG2* 421C>A mutation exerted no affect on the occurrence of CPT-11-induced G4 neutropenia. This correlates with a previous study in Korean patients with non-small cell lung cancer who received CPT-11 + CDDP chemotherapy (21). However, PA317 cells transfected with the *ABCG2* 421C>A mutation show a lower expression of BCRP protein and less drug resistance than wild-type cells, indicating that this mutation changes the phenotype *in vitro* (20). Notably, a case-controlled study of Japanese cancer patients indicated that rs2622604, an SNP in an intron in *ABCG2*, increased the risk of severe myelosuppression due to CPT-11 treatment (31). Therefore, it is important to continue to assess the significance of the variations in *ABCG2* and CPT-11*-*induced neutropenia.

In the present study, an association was demonstrated between the incidence of G4 neutropenia and the UGT1A1*6/*28 or *6/*6 genotype in Japanese patients with gynecological cancers who received low-dose CPT-11 therapy. As the study was retrospective and used a small number of specimens, the additional effect of the UGT1A1*28/*28 genotype could not be investigated. The present study was limited to an investigation of treatment-induced neutropenia and other side-effects, including diarrhea and thrombocytopenia, caused by CPT-11.

Since variants of not only *UGT1A1*, but also other genes, including *UGT1A7*, *UGT1A9*, *ABCB1* and *ABCC2,* have been reported to be involved in the occurrence of CPT-11-induced severe neutropenia (6,11,21,32–35), rare variants of these genes should be investigated in the future. An investigation of the physiological and environmental factors and the risk of severe neutropenia is also required. In addition to age, gender and smoking may also be factors associated with the occurrence of CPT-11-induced severe neutropenia (24,36).

In conclusion, the present study revealed that the UGT1A1*6/*28 and *6/*6 genotypes are associated with the occurrence of severe neutropenia in Japanese patients with gynecological cancer treated with low-dose CPT-11. This finding indicates that the diagnosis of *UGT1A1* variants is as useful for chemotherapy using CPT-11 in gynecological conditions as it is in colorectal and lung cancer patients.

## Figures and Tables

**Table I:** Associations between patient characteristics prior to CPT-11-based chemotherapy and the toxicity outcome of neutropenia.

	G0-3 neutropenia (n=34)		G4 neutropenia (n=10)		
			
Characteristics	Median	Range	Median	Range	P-value^a^
Age, years	55	18–79	59.5	52–72	0.098
Height, cm	153.5	147.0–167.7	151.4	138.5–161.0	0.202
Weight, kg	54.7	38.7–95.7	51.0	42.0–65.5	0.481
BSA, m^2^	1.49	1.27–1.95	1.46	1.24–1.67	0.300
BMI, kg/m^2^	21.8	16.0–40.6	22.4	19.3–28.4	0.933
WBC, mm^3^	4465	2590–8940	4290	3070–7230	0.911
Neutrophils, mm^3^	2590	970–7108	2652	1627–6116	0.737
Total bilirubin, mg/dl	0.52	0.20–1.18	0.48	0.22–1.04	0.889
Albumin, mg/dl	3.9	2.9–4.5	3.9	2.3–4.3	0.966
AST, IU/l	20	12–87	15.5	11–23	0.018
ALT, IU/l	17	5–121	10.5	4–24	0.001
γ-GTP, IU/l	24.2	11–89	15.5	7–35	0.059
ALP, IU/l	245	138–696	238.5	169–376	0.600
SCr, mg/dl	0.67	0.37–1.33	0.645	0.48–0.92	0.793

a Mann-Whitney’s U test;

CPT-11, irinotecan; BSA, body surface area; BMI, body mass index; WBC, white blood cell; AST, aspartate aminotransferase; ALT, alanine aminotransferase; γ-GTP, γ-glutamyltranspeptidase; ALP, alkaline phosphatase; SCr, serum creatinine.

**Table II:** Correlations between the development of grade 4 neutropenia and genotypes, previous treatments, regimens, cancer types and performance status.

	Neutropenia, n (%)		
		
Characteristics	G0-3	G4	P-value^a^
Total patients	34 (77.3)	10 (22.7)	
Genotype			
*UGT1A1*			
Dominant model			0.287
−/−b	21 (84.0)	4 (16.0)	
−/+^c^, +/+^d^	13 (68.4)	6 (31.6)	
Recessive model			0.037
−/−b, −/+^c^	31 (83.8)	6 (16.2)	
+/+^d^	3 (42.9)	4 (57.1)	
*ABCG2* 421C>A			
Dominant model			0.456
C/C	23 (82.1)	5 (17.9)	
C/A, A/A	11 (68.8)	5 (31.2)	
Recessive model			0.120
C/C, C/A	31 (81.6)	7 (18.4)	
A/A	3 (50.0)	3 (50.0)	
Previous treatment			1.000
No	5 (83.3)	1 (16.7)	
Yes	29 (76.3)	9 (23.7)	
Regimen			0.069
CPT-11 + CDDP	20 (90.9)	2 (9.1)	
CPT-11 + MMC	14 (63.6)	8 (36.4)	
Type of cancer			0.147
Ovarian	21 (87.5)	3 (12.5)	
Other	13 (65.0)	7 (35.0)	
Performance status			1.000
0	22 (78.6)	6 (21.4)	
1, 2	12 (75.0)	4 (25.0)	

a Fisher’s exact test;

b *1/*1;

c *1/*6 and *1/*28;

d *6/*28 and *6/*6.

*UGT1A1*, uridine diphosphate glucuronosyltransferase 1A1; *ABCG2*, ATP-binding cassette sub-family G member 2; CPT-11, irinotecan; CDDP, cisplatin; MMC, mitomycin C.
